# Determinants of nondisclosure of HIV status among women attending the prevention of mother to child transmission programme, Makonde district, Zimbabwe, 2009

**DOI:** 10.4314/pamj.v8i1.71169

**Published:** 2011-04-30

**Authors:** Pride Mucheto, Addmore Chadambuka, Gerald Shambira, Mufuta Tshimanga, Notion Gombe, Wenceslas Nyamayaro

**Affiliations:** 1 Department of Community Medicine, University of Zimbabwe, Harare, Zimbabwe; 2 Provincial Medical Directorate, Mashonaland West, Zimbabwe

**Keywords:** Prevention of mother to child transmission, HIV Status, Determinants, Women

## Abstract

**Introduction:**

The 2007 United Nations General Assembly Report on HIV/AIDS in Zimbabwe reported nondisclosure of HIV status as a challenge in the PMTCT programme. Preliminary investigations on nondisclosure among 21 women tested for HIV at Chinhoyi Hospital showed that only six had disclosed their HIV status. We investigated the determinants of nondisclosure of HIV status.

**Methods:**

A cross sectional analytic study was conducted at six health facilities in Makonde district. The Theory of Planned Behaviour was adapted to guide socio-cultural variables assessed. Antenatal and postnatal women tested for HIV in the PMTCT program who consented to participate were interviewed.

**Results:**

We enrolled 334 women. Thirty four percent (114) did not disclose their HIV status. Among HIV positive respondents, 43% (25) did not disclose their status. Women who believed disclosure caused physical abuse (OR=1.81, 95% CI: 1.17-2.90), caused divorce (OR=2.01, 95% CI: 1.25-3.22) and was unimportant (OR= 2.26, 95% CI: 1.33-3.87) were two times less likely to disclose their status. Respondents who received group HIV pre-test counselling were 2.4 times more likely not to disclose. Receiving ANC HIV education at least twice and referral for psychosocial support were significantly protective [OR 0.54 (95% CI 0.24-0.63) and 0.16 (95% CI: 0.06-0.41) respectively. Independent determinants of nondisclosure among HIV positive women were perception that disclosure would cause divorce (AOR=7.82, p=0.03), living with an extended family (AOR=10.3, p=0.01) and needing spousal approval of HIV testing (AOR= 0.11, p<0.001).

**Conclusion:**

Lack of psychosocial support and counselling for women and belief that disclosure causes divorce, abuse or is unimportant contributes to nondisclosure. Identifying women with social challenges and strengthening their referral for psychosocial support can improve disclosure of HIV status and reduce mother to child transmission of HIV.

## Introduction

Human immune deficiency virus (HIV) infection in women of reproductive age fuels the peri-natal HIV epidemic. Without preventive measures the risk of a baby acquiring HIV infection in developing countries is 25 - 45 % [1-3]. The Prevention of Mother to Child Transmission (PMTCT) programme is one of the strategies implemented in the control of the HIV epidemic. Most primary health care facilities in Zimbabwe offer a “minimum package of PMTCT services which consists of providing ARV for prophylaxis, community mobilisation and IEC activities, supportive and follow up counselling and infant feeding counselling. Referral health facilities and a few selected primary health facilities offer a comprehensive package which in addition to the minimum package, provides appropriate training, support and supplies, pre and post test counselling, on site rapid HIV testing and quality assurance, infant diagnosis, monitoring and evaluation and support for different infant feeding options [4].

Disclosure of HIV positive status is a complex, difficult and very personal matter that entails communication about a potentially life threatening, stigmatized and transmissible illness to someone [5]. HIV status disclosure serves as an important prevention strategy in PMTCT, it enables PMTCT attendees to benefit by being able to use ART prophylaxis, practice safe infant feeding and family planning practices [3,4]. Disclosure is also important for psychosocial support, treatment adherence [6-8], stigma reduction and risk reduction behaviour [9] In developed countries HIV status disclosure rate among antenatal care (ANC) women ranges from 42-100% compared to 16.7-32% for developing countries [10]. The Policy in Zimbabwe advocates that all patients and clients, negative and positive, should be empowered to disclose their results to their sexual partners through counselling.

Studies show that disclosure of HIV status is influenced by a number of factors. It depends on whether partnership is regular, close, main compared to casual and unfamiliar relationship [11], person being disclosed to has a known positive status [12-16], number of lifetime sex partners [17], social factors (support [18], fears of abandonment, discrimination, violence and accusations of infidelity) [19-23], individual self efficacy [24], education [16,25], gender [26], marital status [27], illness severity and length of time since diagnosis [11,28].

The HIV prevalence among pregnant women is 13.1% for the 15-24 year olds and 17.7% for the 25-49 year olds in Zimbabwe [29]. The PMTCT package offered in Zimbabwe is consistent with WHO guidelines for 2006. The PMTCT programme was first implemented in Zimbabwe under the national HIV and AIDS Policy of 1999, with services being integrated into the antenatal care services thereafter [29,30]. By 2006, 98% of health facilities, a total of 1422 facilities in the country were offering PMTCT services. Five hundred and forty seven facilities offered “comprehensive PMTCT services”, which include HIV testing and counselling and ARV prophylaxis onsite, the rest of the facilities only offered supportive counselling and antiretroviral (ARV) prophylaxis without on-site HIV testing [31]. The programme has challenges among them technical challenges, limited human resources capacity, low male participation in HIV testing and counselling and poor disclosure of HIV status [32].

The 2007 United Nations General Assembly Zimbabwe’s country report on HIV and AIDS stated poor disclosure of HIV status by women to significant others (husband/partner, in-laws, siblings or friends) as one of the major challenges in the PMTCT programme [32]. Makonde District has urban, peri-urban (mining) and rural populations (communal, resettlement and commercial farming). The district caters for a population representative of most districts within the country. The PMTCT programme in Makonde District attended to 1368 pregnant women and 1230 of them were counselled, tested for HIV and reported 175 HIV positive mothers to have been referred to the local psychosocial support groups in 2008. Twenty three (13.1%) of the 175 referred pregnant women opted to enrol for psychosocial support and counselling at the local support group. The support groups offer post test counselling, health education, and support mechanisms for disclosure of HIV. In April 2009 at Chinhoyi Provincial Hospital, preliminary investigations conducted on nondisclosure of HIV status among 21 women who had undergone testing for HIV in the PMTCT programme showed that only six of them had disclosed their HIV status to significant persons among them spouses. With this background we identified the factors leading to nondisclosure of HIV status among PMTCT attendees in Makonde district.

## Methods

**Design**

A cross sectional analytic study was conducted

**Setting**

Six health facilities offering comprehensive PMTCT in Makonde District of Mashonaland West, Zimbabwe.

**Study participants**

Antenatal and postnatal women attending ANC or postnatal care (PNC) follow up visits during the study period and were tested for HIV in the PMTCT programme were enrolled.

**Sampling and sample size**

We used Epi-Info statistical software to determine sample size. Using poor relationship with spouse as our exposure of interest and assuming an expected frequency of nondisclosure of HIV status in the unexposed group (those with a good relationship with spouse) of 20% and 34.5% in the exposed group (those with poor relationship with spouse), based on the Kenyan study [33], we calculated a sample size of 320 participants (at 95% confidence interval and 80 % power). Assuming an 80% response rate, the minimum sample size needed was of 400 participants. All mothers attending ANC and PNC clinics during the period May to June who had consented to an HIV test in the Makonde PMTCT programme were eligible to participate. We recruited all mothers during that period and recruitment was done from Monday to Friday at all facilities by trained research assistants

**Data collection tools and methods**

Two focus group discussions were conducted using a focus group discussion guide, one with 11 pregnant women and another with 10 postnatal care mothers, to identify locally applicable behavioural, normative, and control beliefs most commonly held on PMTCT. We used the beliefs identified to develop constructs (group of questions or scaled items) using the Theory of Planned Behaviour (TBP) as a basis to explain the behaviour of disclosure. The TBP is a model applied to understand behaviour. It suggests that proximal determinants of behaviour are one’s intention to engage in that behaviour and one’s perceptions of control over the behaviour. Intention is determined by three sets of factors (attitudes, subjective norms and perceived behavioural control). According to TBP, individuals are likely to follow a particular health action if they believe that the behaviour will lead to outcomes which they value, if they believe the people whose views they value think they should carry out the behaviour and if they have the necessary resources and opportunities to perform the behaviour [34]. The constructs were incorporated into the interviewer administered questionnaire. The questionnaire elicited demographic factors, previous HIV screening, knowledge on importance of disclosure, relationship and social factors and assessed these variables for their association with the outcome.

**Reliability of constructs subscales**

The Theory of Planned Behaviour Model constructs were tested for reliability before they were used. All constructs with subscales except for behavioural beliefs had Cronbach’s alphas above the acceptable level of 0.7.

**Pre-testing**

The questionnaire was pre tested at Banket District Hospital.

**Data analysis**

Quantitative data was processed and analyzed using Epi info version 3.01 statistical package. Information on variables such as demographic factors and knowledge was summarized and reported as frequencies and proportions. Bivariate analysis was conducted to calculate odds ratios and 95% confidence intervals. Multivariate analysis using stepwise logistic regression was done for the factors found with a p-value less than 0.25 in the bivariate analysis to determine the independent predictors of non disclosure. Correlation analysis was conducted using STATA statistical software package to assess correlations between the scales in each of the constructs and between constructs. STATA was also used to determine the Cronbach alphas for the scales. The analysis of qualitative data was done manually. Data generated from open ended questions was categorized and reported as proportions and frequencies for the categories of responses given.

**Ethics and permissions**

We obtained ethical clearance from the Parirenyatwa Hospital Institutional Ethical Review Board. Permission to carry out the study was obtained from the Provincial Medical Director, Health Studies Office. Informed written consent was obtained from participants and confidentiality assured and maintained. No names were captured on the questionnaires. The investigators counselled the participants individually after the interview session where it was possible and referred other respondents for further individual counselling by program counsellors.

## Results

**Analytic study findings**

Three hundred and thirty four respondents were enrolled into the analytic study. Table 1 summarizes the demographic characteristics of the participants. Thirty four percent (114) of the participants in the study had not disclosed their HIV status.

Slightly above half of women 55.7% (34) with spouses who had less than seven years of education did not disclose their HIV status compared to 29.3% (80) women with spouses with more than seven years of education. There was no significant difference in the proportions of married women (32.7%, 96) and cohabiting women (35.7%, 5) who did not disclose their HIV status. Women who disclosed their HIV status and those who did not had similar median years in marriage and they had a median parity of one child (Q1= 0, Q3=2). Women reporting HIV status disclosure disclosed their status to either partners/spouses, siblings, friends, parents or in-laws.

**HIV testing experience**

Other than being tested for HIV in the PMTCT programme, 49% (164) of all the respondents had undergone HIV testing before. Forty seven percent (53) of respondents who had not disclosed and 51% (111) of those who had disclosed their status had undergone HIV testing before. More than 55% of those tested for HIV before did so in the voluntary counselling and testing facilities. Thirty six percent (41) of the women who had not disclosed had known their HIV status for duration of less than 3 months compared to 15% (33) of those who disclosed their HIV status.

**Level of knowledge on the importance of HIV status disclosure**

There was no difference in the level of knowledge on the importance of disclosure of HIV status in PMTCT interventions between respondents who did not disclose (82.5%, 94) and those who disclosed (89.1%, 196), p-value of 0.08 for all concepts assessed. Seventy six percent (87) of the respondents who did not disclose and eighty four percent (184) of those who disclosed reported that nondisclosure of one’s HIV status hinders access to PMTCT care.

**Nondisclosure of HIV status among PMTCT attendees**

Seventeen percent (58) of all respondents were HIV positive and 83% (276) were negative. Forty five percent (26) of HIV positive respondents did not disclose their HIV status compared to 32.2% (88) HIV negative respondents. Forty four percent (11) positive women reported they did not disclose because their partners were uncooperative, thirty percent (27) of negative women reported they did not disclose their results because they felt the results were their secret. Other reasons reported for non disclosure were that the women felt it was not necessary to disclose and they were still deciding on when to do so. Most women, 79.5% (175) reported having disclosed their status within a few days after being tested, 1.8% (2) disclosed when they were deciding on delivery method, 19.8% (40) were tested for HIV with their partner or spouse in the PMTCT programme.

**Risk factors for nondisclosure of HIV status among PMTCT attendees**

Respondents who resided in rural settings were two times more likely not to disclose their HIV status to any significant relation compared to those residing in urban settings. No item assessing perceived behavioural control was associated with nondisclosure of HIV status. Respondents with behavioural beliefs that disclosure may cause divorce or physical abuse were two times more likely not to disclose their HIV status. Table 2a and Table 2b summarize the risk factors for nondisclosure of HIV status among women in the Makonde PMTCT programme.

We controlled for HIV status as a confounder in the relationship between having been referred for psychosocial support and nondisclosure of HIV status through stratified analysis (adjusted OR= 0.08, 95% CI 0.03-0.26). HIV positive women who had been referred for psychosocial support were 92% more likely to disclose their HIV status than those not referred for psychosocial support. We observed interaction between HIV status and the normative belief that valuable people approve of the woman’s HIV status disclosure and therefore reported stratum specific OR (Table 2b) (chi square =10.99, p= 0.009).

**Independent determinants of nondisclosure of HIV status among PMTCT attendees, Makonde District, 2009**

Based on literature which states that disclosure is more likely if a person has an HIV negative result as compared to a positive result, and the interaction due to HIV status identified in stratified analysis, we assessed for independent determinants of nondisclosure for HIV positive and negative women separately using the step up procedure for logistic regression model building. Table 3a and Table 3b summarize the independent determinants.

**Intention to disclose HIV status**

Subjective norms in the TPB were the only construct significantly correlated with intention (r=0.24, pP0.05). Twenty six percent (23) of HIV negative women who had not disclosed had no intentions of disclosing their HIV status in the following one year, compared to 38% (10) HIV positive women. Of the 114 women who did not disclose their HIV status, 70% (80) of them reported preferring to disclose their HIV status to their spouses. Thirty four women who reported preferring not sharing their HIV status with anyone, of these 35% (12) of them were HIV positive and 65% (22) were HIV negative.

**Focus group discussion findings**

Twenty one women, eleven pregnant and ten postnatal participated in the focus group discussions. All of them had been tested for HIV in the PMTCT programme. The median parity of the participants was one child and the mean age was 27years. All participants were in agreement that the incorporation of PMTCT in ANC has reduced stigma and there is no segregation of those found positive, however the approach resulted in the programme being gender biased, with minimal spousal or partner involvement. The participants reported that the programme’s emphasis is on the unborn baby’s benefits and women are left with no choice. Pregnant participants said “If you are pregnant the health workers tell you, you cannot get medical treatment unless you get tested for the child’s protection”. One postnatal woman said, “”When I was instructed by health workers to bring my spouse for testing, my husband asked me, ’Am I pregnant too, why should I come along?’ Another one said, ”The biggest challenge we have as women is that it is difficult for us to initiate the discussion on HIV testing. Men tend to become violent and think we suspect them of being promiscuous. This makes communication on this subject very challenging.” One prime-up antenatal participant said, ”My husband refused to get tested to whom do you expect me disclose my status. He will divorce me.”

Stigma on HIV issues is still high within the community. The community is still not accepting some PMTCT interventions such as exclusive breastfeeding. HIV negative women felt vulnerable because once tested negative health workers seem not to do much for them, they are left with not enough support and information yet there is still a chance of infection and transmission of the virus to the unborn baby. A significant proportion of the participants (8/11 pregnant women and 7/10 postpartum mothers) reported having not been referred for any form of support after being tested. All participants reported having been encouraged to disclose their status by the health staff. However they doubted if there was strict confidentiality of their results.

## Discussion

Nondisclosure of HIV status was higher among HIV positive respondents (45%) compared to those negative (32%) among PMTCT attendees in Makonde district. The rate of nondisclosure among HIV positive respondents in our study was similar to the rate noted in Kenya where 31% of women in VCT and PMTCT programs failed to disclose their HIV positive status [33]. In a PMTCT program in Uganda, it was noted that a substantial proportion (75%) of HIV positive women in the PMTCT program did not disclose their result to their partners [35]. This is in contrast to findings in south west Ethiopia and northern Nigeria where disclosure was 94.5% and 89% respectively [36-38] HIV status disclosure for positive people though beneficial has psychosocial implications attached which often deter positive people from disclosing [4,39, 40].

Regardless of HIV status, disclosure is of importance in PMTCT programmes as it allows an individual to get spousal or family support for preventive actions they may decide to undertake. Among negative women disclosure may motivate the sexual partner to seek testing and among positive women it enables couples to make informed reproductive health choices [19]. A cohort study in Abidjan Côte d’Ivoire, nearly all negative (97%), but less than half (46%) positive women disclosed their HIV status at the end of follow up, where psychosocial support was offered to women. The cohort was conducted in research PMTCT programme hence the higher disclosure rates [41]. Failure to disclose by negative women and the low male participation in our study, may hinder efforts by the programme to emphasize the understanding of discordant couples and need to encourage partner testing as it is possible that even though the woman is negative, the partner could be infected and therefore transmission can still occur to the unborn child [32].

Behavioural beliefs that disclosure may cause divorce or physical abuse by spouse were risk factors for nondisclosure, having a normative belief that a spouse or partner expects them to disclose and subjective norm that most important people approve of the women to disclose their status were significantly protective. The results support the finding observed in Tanzania where it was noted that there was need to consider the social context of HIV status disclosure, as social environment shapes the process of status disclosure [42]. The beliefs that disclosure may result in divorce or physical abuse identified in our study might form useful targets for interventions to improve disclosure of HIV status. Similar findings were obtained in Ethiopia where women did not disclose because they were concerned about divorce/separation, physical attacks and acts of murder as a result of disclosure [43] and in South Africa [44-46]. Interventions may also be directed towards strengthening the normative beliefs and the subjective norms, in which women felt disclosure as acceptable in the family environment and hence the likelihood to disclose.

Group counselling was done during HIV pre-test counselling in Makonde. The current severe understaffing in health facilities has seen group talks and pre-test counselling being adopted to circumvent the shortage and to reduce the counselling burden on health workers [46]. Considering that slightly above half (51%) of participants had their first HIV test in the PMTCT programme, group counselling might hinder individuals from verifying personal concerns during sessions as there is no confidentiality in these sessions hence the likelihood of not disclosing among those who had group counselling. Health facilities have been noted to be the first source of information for HIV issues for slightly above half (52%) of PMTCT women attending health facilities, the use of group counselling may hinder the effort to offer the information effectively [47]. Group counselling was noted not to pay attention to individual problems and circumstances or maintain confidentiality. Key to disclosure of HIV status by women is prior communication with partners or spouses on the subject of testing. Studies have shown that disclosure is higher where there was prior discussion [43].

Having known one’s status for less than 3 months was an independent significant predictor for non disclosure. Women who had a short duration of knowing their HIV status were more likely not to disclose their HIV status. In a Kenyan study, women with long duration of knowing their HIV status were more likely to disclose their HIV status. In Northern Nigeria, 45.6% of HIV positive women took more than a year to disclose their status [37]. Long duration enables women to adjust to their result especially if the result is positive [33]. This contrasts with findings in Abidjan where two thirds were reported to have disclosed before delivery as they were influenced by feeding options that they intended to use [41].

In logistic regression having stratified our data by HIV status, independent determinants for non disclosure for positive women were, perceiving disclosure as likely to cause divorce, living with an extended family and spousal approval. In Kenya, divorce was noted to be a barrier to disclosure of HIV status mainly due to economical reasons and social implications. Considering that 90% (103) of those who did not disclose in Makonde were not employed, HIV positive women probably were more likely not to disclose due to the fear of economic losses associated with divorcing [33]. Rates of disclosure may be associated with expectations of social support [39].

Women living with extended families were two times more likely not to disclose their HIV status. Conjugal confidentiality was found to be an important determinant of HIV status disclosure regardless of the HIV status [41]. Extended families by virtue of set up of sharing a house with other relations may pose challenges in communication between partners as the living conditions may not be suitable for conjugal confidentiality. Disclosure can be selective, where participants disclose to some relationship members and not to others [39]. Disclosure reported in this study was to partner/spouse, siblings, friends and parents.

**Limitations**

The study was based on interview information, with the outcome variable of nondisclosure being assessed based on self reporting by study participants, this may likely to have resulted in recall bias particularly for postnatal women. Over estimation or underestimation of the prevalence of nondisclosure cannot be ruled out as information obtained could not be verified if the women had actually disclosed. The findings could also have been biased as participants might have reported what they felt the health worker conducting the interview expected. Results of the qualitative analysis might have been affected by skills of the researchers which may not have been equally good, the inherent fact that rigor of analysis is difficult to maintain and that data may not be generalized to the general population as it was only from a few individuals.

## Conclusion

Independent factors for non-disclosure among HIV negative participants were mainly social and health service related factors. Addressing the social and health service factors may significantly impact on disclosure in this group. Among the HIV positive participants, independent determinants were mainly social factors and therefore to improve disclosure, interventions should focus on the social aspects. Addressing normative and behavioural beliefs is equally important in both HIV positive and negative people. Referral of individuals to psychosocial support groups by health workers and participation in groups activities remains important for disclosure of HIV status.

We recommend that counselling for PMTCT focus on dealing with social factors and normative and behavioural beliefs that impact on disclosure. Management should address health system factors that result in non-disclosure of HIV status. Improvement in referral of PMTCT attendees for psychosocial support through strengthening of the communication between the PMTCT program and locally available psychosocial support groups and where they are not available facilitate the formation of support groups is recommended. Mechanisms to ensure individual pre-test counselling to PMTCT attendees should be devised as this may identify patients having social challenges and enable assistance to be offered accordingly. Regular health education on the importance of partner participation in the PMTCT program must be given to improve male participation in the PMTCT programme. Program counsellors should discuss the importance of disclosure often and directly during counselling sessions.

A follow up study on the determinants of male partner participation was conducted based on our findings. A PMTCT support group was formed for Chinhoyi Provincial Hospital PMTCT programme.

## Competing interests

The authors declare no competing interests.

## Authors’ contribution

Mucheto P: was responsible for the conception of the problem, design, collection, analysis and interpretation of data and drafting the final article, Chadambuka A: was responsible for the conception of the problem, design, analysis and interpretation of data and drafting and critically reviewing the final article, Shambira G: was responsible for the conception of the problem, design, collection, analysis and interpretation of data and drafting the final article, Tshimanga M: Had oversight of all the stages of the research. Critically reviewed the final draft for academic content, Gombe N.T: Participated in the design, analysis and interpretation of data and drafting the final article, Nyamayaro W: was responsible for the design, analysis and interpretation of data and drafting the final article. All the authors have read and approve the last version of the manuscript.

## Acknowledgements

We acknowledge the assistance we got from Mrs J. Chideme-Maradzika on how to apply the Theory of Planned Behaviour in our study.

## Figures and Tables

**Table 1:** Demographic Characteristics of Women Attending PMTCT in Makonde District, 2009

Characteristic	Total (N)	Not disclosed n(%)	Disclosed n(%)	p-value
**Woman’s employment status**				
Employed	47	11 (23.4)	36 (76.6)	0.063
Unemployed	287	103 (36)	184 (64)	
**Level of education of woman’s spouse**				
< 7 years of education	143	61 (42.7)	82 (57.3)	0.003
> 7 years of education	191	53 (27.7)	138 (72.3)	
**Woman’s area of residence**				
Rural	117	54 (46.2)	63 (53.8)	<0.001
Urban	217	60 (27.6)	157 (71.3)	
**Woman’s religion**				
Apostolic	95	45 (47.4)	50 (52.6)	0.342
Orthodox	100	29 (29)	71 (71)	
Pentecostal	102	27 (26.5)	75 (73.5)	
Other	37	13 (35.1)	24 (64.9)	
**Woman’s age**				
Median age (Q_1_:Q_3_)		24 (21:30)	26 (22:32)	

**Table 2a:** Factors Associated with Nondisclosure of HIV Status by Women in Makonde District PMTCT Programme, 2009

Factor	Not Disclosed n = 114	Disclosed n = 220	Odds Ratio (95% CI)
Woman resides in rural area	54	63	2.24
Woman resides in urban area	60	157	(1.40–3.59)
Woman’s spouse has < 7 years education	61	82	1.94
Woman’s spouse has >7 years education	53	138	(1.23–3.06)
Woman’s spouse/partner unemployed	48	44	2.91
Woman’s spouse/partner employed	66	176	(1.77–4.78)
Woman living with extended family	48	65	1.73
Woman not living with extended family	66	155	(1.08–2.78)
Woman perceived disclosure is unimportant	35	36	2.26
Woman perceived disclosure is important	79	184	(1.33–3.87)
Woman perceived disclosure causes divorce	77	112	2.01
Woman perceived disclosure doesn’t cause divorce	37	108	(1.25–3.22)
Woman perceiving disclosure causes physical abuse	64	90	1.81
Woman perceiving disclosure doesn’t cause physical abuse	50	130	(1.17–2.9)
Woman attended group HIV counselling	88	129	2.39
Woman didn’t attend group HIV counselling	26	91	(1.43–3.99)
Woman knew HIV status for <3months	25	33	3.18
Woman knew HIV status for >3months	89	187	(3.19–5.49)

**Table 2b:** Factors associated with nondisclosure of HIV status by women in Makonde district PMTCT Programme, 2009

Factor	Not Disclosed n = 114	Disclosed n = 220	Odds Ratio (95% CI)
Woman received HIV health education > twice	31	108	0.38 (0.24–0.63)
Woman received HIV health education < twice	83	112	
Woman offered psychosocial support	5	49	0.16 (0.06–0.41)
Woman not offered psychosocial support	109	171	
Woman perceiving most people approve disclosure	53	143	0.47 (0.3–0.74)
Woman perceiving most people disapprove disclosure	61	77	
Woman’s spouse expects disclosure	71	162	0.59 (0.36–0.96)
Woman’s spouse doesn’t expect disclosure	43	58	
Woman had HIV ANC health education	82	182	0.54 (0.31–0.92)
Woman had no HIV ANC health education	32	38	
Woman’s spouse attends ANC/PNC	9	76	0.16 (0.08–0.34)
Woman’s spouse doesn’t attends ANC/PNC	105	144	
**HIV positive women**			
Most people approve disclosure	5	26	0.07* (0.02–0.24)
Most people disapprove disclosure	109	194	
**HIV negative women**			
Most people approve disclosure	48	117	0.70* (0.42–1.17)
Most people disapprove disclosure	66	103	

* Stratum specific odds ratios

**Table 3a:** Independent Determinants of Non-disclosure of HIV Status among PMTCT attendees, Makonde District, Zimbabwe, 2009

Characteristic	All Participants			HIV Negative Participants				HIV Positive Participants			
	Not Disclosed	Disclosed	OR (95% CI)	Not Disclosed	Disclosed	OR (95% CI)	AOR (95% CI)	Not Disclosed	Disclosed	OR (95% CI)	AOR (95% CI)
**Social Factors**											
Woman’s spouse attend ANC/PNC											
Yes	9	76	0.16	6	66	0.13	0.19	3	10	0.31	0.06
No	105	144	(0.08–0.34)	83	121	(0.06–0.32)	(0.08–0.43)	22	23	(0.08–1.29)	(0.03–1.02)
Woman’s spouse approves HIV testing											
Yes	64	117	1.13	50	140	0.43	0.32	5	25	0.13	0.11
No	50	103	(0.72–1.78)	39	47	(0.25–0.73)	(0.13–0.44)	20	11	(0.04–0.42)	(0.02–0.5)
Woman’s spouse has <7years of education										
Yes	34	27	3.04	30	19	4.5	2.15	1	7	0.15	0.04
No	80	193	(1.74–5.36)	59	168	(2.35–8.58)	(1.15–4.02)	24	26	0.02–1.35	(0.00–3.3)
Woman resides with extended family										
Yes	48	65	1.73	36	60	1.4	1.20	12	5	5.17	10.3
No	66	155	(1.08–2.78)	53	127	(0.85–2.43)	(0.64–2.20)	13	28	(1.51–17.7)	(1.6–66.0)
**Normative beliefs and behavioural beliefs**											
Woman’s spouse approves disclosure											
Yes	71	162	0.59	48	117	0.7	–	5	20	0.07	–
No	43	58	(0.36–0.96)	41	70	0.42–1.17		20	7	(0.02–0.24	
Woman believes disclosure causes divorce										
Yes	77	112	2.01	67	98	5.07	3.48	10	14	0.9	7.82
No	37	108	(1.25–3.22)	12	89	(2.47–10.6)	(1.74–6.89)	15	19	0.28–2.96	(1.16–53.0)

OR: Odd Ration, AOR: Adjusted Odd Ratio

**Table 3b:** Independent Determinants of Non-disclosure of HIV Status among PMTCT attendees, Makonde District, Zimbabwe, 2009

Characteristic	All Participants			HIV Negative Participants				HIV Positive Participants			
	Not Disclosed	Disclosed	OR (95% CI)	Not Disclosed	Disclosed	OR (95% CI)	AOR (95% CI)	Not Disclosed	Disclosed	OR (95% CI)	AOR (95% CI)
**Health services factors**											
Woman received group HIV counselling											
Yes	88	129	2.39	47	82	1.40	2.05	8	7	1.75	0.73
No	26	91	(1.43–3.99)	42	105	(0.8–2.38)	(1.07–3.93)	17	26	(0.50–5.71)	(0.07–7.07)
Woman offered psychosocial support											
Yes	5	49	0.16	53	64	2.83	0.08	16	22	0.89	8.65
No	109	171	(0.06–0.41)	36	123	(1.68–4.76)	(0.01–0.52)	9	11	(0.30–2.65)	(0.93–80.9)
Woman received health education on HIV >twice											
Yes	31	108	0.38	20	89	0.32	0.36	11	19	0.58	0.12
No	83	112	(0.24–0.63)	69	98	(0.18–0.57)	(0.21–0.8)	14	14	(0.21–1.60)	(0.01–1.24)
**Other factors**											
Woman had knowledge of HIV status <3 months											
Yes	25	33	3.18	36	28	3.86	3.63	5	5	1.4	16.0
No	89	187	(3.11–5.49)	53	159	(2.15–6.91)	(1.29–6.37)	20	28	(0.3–5.40)	1.10–231.5

OR: odd ration, AOR: Adjusted Odd ration^i^
